# Prevalence of HIV infection and uptake of HIV/AIDs services among fishermen on the shores of Lake Victoria in Kagera region, Northwestern Tanzania

**DOI:** 10.1371/journal.pone.0315265

**Published:** 2025-01-24

**Authors:** Patrick Mwanahapa, Mtoro J. Mtoro, Dina Gerald, Pius Horumpende, Salaam Mujeeb

**Affiliations:** 1 Nexus International University, Kampala, Uganda; 2 Global Programs, Dar es Salaam, Tanzania; 3 Cavendish University, Kampala, Uganda; 4 Ministry of Health, Dodoma, Tanzania; 5 Islamic University in Uganda, Kampala, Uganda; 6 Mount Kenya University, Thika, Kenya; Government College of Engineering, Keonjhar, INDIA

## Abstract

**Introduction:**

The Tanzania HIV Impact Survey (THIS) 2022–2023 showed that HIV prevalence among the general population stabilises but varies geographically across the country. Despite this, disproportionate burdens of HIV continue among specific subpopulations, such as fishermen. Fishermen are particularly vulnerable to HIV infection and have a low uptake of HIV prevention and treatment services. This study aimed to understand the prevalence of HIV infection, uptake of HIV/AIDs services, and associated risk factors of HIV Infection among fishermen residing along the shores of Lake Victoria in the Kagera region, Tanzania, in 2024.

**Methods:**

A cross-sectional study among fishermen was employed from February to April 2024, using convenient sampling of 10 beach management units (BMUs) to obtain a robust sample of fishermen aged ≥15 years in Kagera. Participants were randomly selected across BMUs. Information was collected using an interviewer-administered questionnaire, and HIV testing was offered on-site according to national testing guidelines. Multivariable logistic regression was used to determine factors associated with HIV infection, adjusted for potential confounders.

**Results:**

A total of 774 fishermen with a median age of 31 years (interquartile range: 25–38 years) were recruited. The study found an HIV prevalence of 11.3% (95% CI: 9.2–13.8). HIV prevalence varied across selected districts: 12.7% in Muleba District, 10.1% in Bukoba Rural District, and 8.6% in Bukoba Urban District. The HIV prevalence was highest among fishermen aged 20–24 years (14.7% (95% CI: 9.7–21.5). The majority, 60.9% (95% CI: 57.4–64.2), had tested for HIV in the past 12 months. Of those living with HIV, 77.6% (95% CI: 67.3–85.9) self-reported using anti-retroviral therapy. In adjusted analysis, using alcohol before sex (aOR = 2.32, 95 CI: 1.42–3.80), not testing for HIV in the last 12 months (aOR = 4.69, 95% CI: 2.79–7.88), and not using condoms (aOR = 1.94, 95% CI: 1.13–3.27) were significantly associated with HIV infection among fishermen.

**Conclusion:**

HIV prevalence among fishermen was nearly twice as high as in the general population in Kagera. HIV programming should be strengthened to reduce new HIV infections. Hotspot mapping to expand HIV prevention and treatment services is pivotal for controlling the HIV epidemic.

## Introduction

In 2022, there were about 39 million HIV-positive individuals worldwide; of these, 37.5 million were adults, and 1.5 million were children under 15 years old [1]. Eastern and Southern Africa reported approximately 20.6 million HIV- positive individuals from the period 2010 to 2021 [2]. In 2022/2023, there were nearly 1.5 million HIV-positive adults (15 years and above) in Tanzania. However, 82.7% knew their HIV status, and 97.9% of those aware of their HIV status were receiving antiretroviral therapy (ART) [3].

According to the World Health Organization (WHO), males are a crucial demographic to reach with novel HIV testing programs and treatment enrolment [4]. Due to the lower uptake of HIV/AIDS services among men compared to women, men are performing worse than expected in terms of the UNAIDS 95-95-95 targets. In Tanzania, by 2022/2023, 78.4% of men knew their HIV status, compared to 84.8% of women; 96.7% of men were receiving care and treatment compared to 98.4% of women; and 92.9% of men were virally suppressed, compared to 94.9% of women [3].

Since the first known case of HIV in Tanzania occurred in the Kagera region in 1983, the virus has continued to be a significant issue in that area [5]. By 2022/2023, Tanzania’s HIV prevalence was reported to be 4.4%, while the Kagera region had an HIV prevalence of 5.7%. The region was among the top five regions with a higher HIV prevalence (Njombe Region 12.7%, Iringa Region 11.1%, Mbeya Region 9.6%, Songwe Region 6.0%, and then Kagera Region) [3] with the prevalence significantly influenced by fishing communities, including fishermen [6]. Despite a decline in HIV prevalence in Tanzania among the General population from 7% in 2003/2004 [7], 5.1% in 2011/2012 [8], 4.7% in 2016/2017 [9], and 4.4% in 2022/2023 [3], subpopulations such as fishermen have an HIV prevalence above 10%, which is higher than that of the general population [10]. This highlights a significant public health issue, particularly focusing on the prevalence of HIV among this at-risk population—fishermen in Tanzania’s Kagera region.

Fishing is seasonal; markets and fishermen frequently switch between fishing locations. This roving lifestyle poses a significant barrier to enrolment and retention in HIV services [11]. Habitually untreated fishermen are more likely to transmit HIV to their counterparts due to risky behaviours such as unprotected sexual intercourse [12] and a lack of awareness about accessing HIV/AIDS services. This underscores the need for extensive HIV interventions for fishermen due to the high HIV burden. In Tanzania, the fishing industry employs 30,064 aquafarmers and 195,435 fishers directly, contributing 1.7% of the Gross domestic product (GDP). Furthermore, approximately 4.5 million individuals (6.89% of the total population) work in various supplementary roles throughout the two value chains [13]. Therefore, implementing interventions to prevent HIV transmission is crucial, as HIV/AIDS can significantly impact national development when fishing communities are affected without early and continued treatment.

Elsewhere, studies among fishermen reported an HIV prevalence of 23.3% in Kenya [14] and 17.5% in Uganda [11], attributed to the low uptake of HIV services. Additionally, high mobility and a lack of care and treatment facilities have been identified as the main barriers to the utilisation of HIV services, including ART uptake and HIV testing. [15, 16] contributing to the high HIV prevalence. A meta-analysis conducted in Africa and Asia [12] found a low condom uptake of 48% among fishermen during sexual intercourse, which leads to an ongoing risk of HIV transmission among these priority populations in Tanzania.

There is scarce evidence or research done in Tanzania regarding the progress of HIV prevalence and ongoing risk factors of HIV Infection among fishermen living along the shore of Lake Victoria in the Kagera region, as the epidemiology of HIV infection among fishermen was last studied in 2017 [10]. Therefore, this study aims to assess the progress in addressing HIV infection among fishing communities. The objectives are to estimate HIV prevalence, understand the extent of HIV/AIDS services uptake, and identify associated risk factors of HIV Infection among fishermen residing along the shore of Lake Victoria in three districts: Muleba District, Bukoba Rural District (RD), and Bukoba Urban District (UD) in the Kagera region.

## Methods

### Description of the study area

This study was conducted in Northwestern Tanzania, in the Kagera region on the shores of Lake Victoria. The survey included three districts: Bukoba Rural District (RD), Bukoba Urban District (UD), and Muleba District. The Kagera region comprises eight districts: Bukoba RD, Bukoba UD, Muleba District, Biharamulo District, Kyerwa District, Ngara District, Missenyi District, and Karagwe District. The region has an estimated population of 2,989,299 according to the 2022 Census, with a 2.0% annual population change from 2012 to 2022 [17].

**Fig 1:**
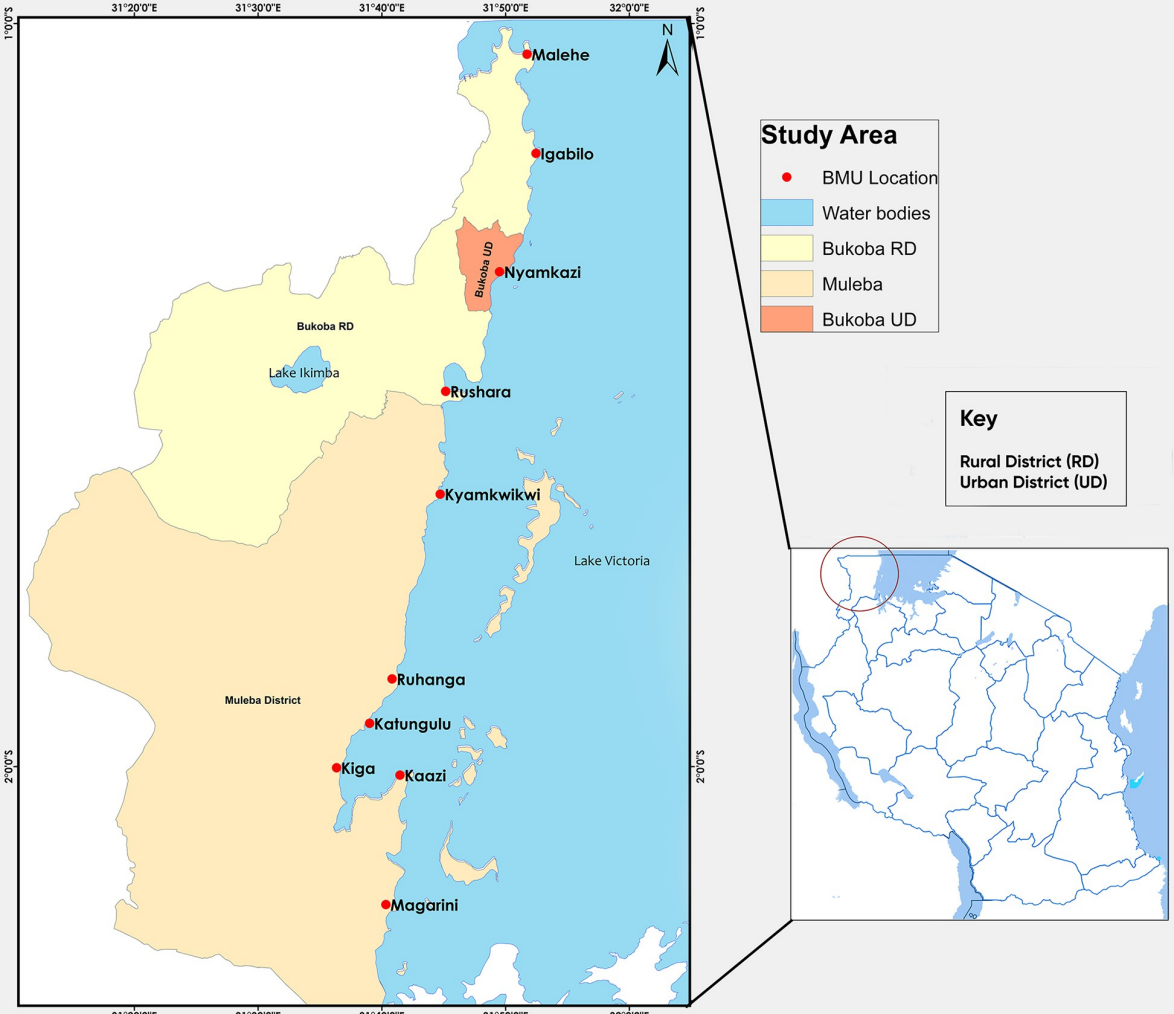
A map showing the location of the Beach Management Units (BMUs) in the three selected districts along the shore of Lake Victoria where the study participants were found during the study period. The Map was drawn using ArcGIS desktop software version 10.8. The shapefiles used were from an openly available source (https://www.nbs.go.tz/statistics/topic/gis), and modifications have been made to capture the study areas.

Muleba District covers an area of 3,518 km^2^, Bukoba RD covers 1,618 km^2^, and Bukoba UD covers 84.25 km^2^, all bounded by Lake Victoria. The study was conducted across ten Beach Management Units (BMUs) located along the shores of Lake Victoria. The following BMUs were included in the study: Malehe, Igabilo, and Rushara (Bukoba RD). Nyamkazi (Bukoba UD). Kyamkwikwi, Ruhanga, Katungulu, Kiga, Kaazi, and Magarini (Muleba District), as shown in **Fig 1**.

### Study design and study population

This study was an analytical cross-sectional study that employed a quantitative data collection method. The recruitment period for the study participants started on 29 February 2024 and ended on 31 April 2024.

- **Inclusion criteria:** This quantitative approach included all fishermen aged above or equal to 15 who resided along the shores of Lake Victoria during the study period.
- **Exclusion criteria:** This quantitative approach excluded all fishermen aged below 15 who resided along the shores of Lake Victoria during the study period and those who didn’t want to sign the informed consent forms.

### Sampling, sample size estimation, and participant recruitment

Beach Management Units (BMUs) are landing sites for fish mongering, boating, and net repair and also include guesthouses, bars, and other services for fishermen. All shores where fishermen are found are identified as BMUs. A list of all BMUs was obtained from local and BMU leaders. Convenience sampling was used to select BMUs along the shores of Lake Victoria in Bukoba RD, Bukoba UD, and Muleba District based on the high number of study participants, availability and geographically proximity. Key informants, who were fishermen living on the shore of Lake Victoria but not part of the study population, assisted in selecting BMUs. The key informants only provided information to the research assistants.

A list of households within the BMUs was obtained from the local and BMU leaders, and a systematic sampling approach was applied, selecting every 25th household from the list after randomly choosing a starting point. In each household, study participants were randomly selected to be part of the study.

The sample size was calculated using a sample size calculator [18] with a 95% confidence interval, 50% population proportion, and a margin of error of 0.05. A total of 878 fishermen were approached, and 774 who voluntarily agreed to participate were included in the estimated sample size for the community-based survey.

### Data collection

A structured questionnaire was used to collect information on participant demographics and the uptake of HIV/AIDS services among fishermen residing along the shores of Lake Victoria in the Kagera Region. The questionnaire gathered data on social and demographic characteristics, risky behaviours, HIV/AIDS services uptake, HIV prevalence, and factors associated with the risk of HIV infection among fishermen. The questionnaire (**S1 Appendix)** was adopted from a published study [10] It was then modified according to this current study requirement. Before collecting the data, the questionnaires were validated by performing a pilot study among participants residing on the shores of Lake Victoria.

Purposive sampling was used to select research assistants (health professionals) and key informants involved during the study period. Key informants were chosen based on the area having a high number of study participants. Research assistants, including nurses and clinical officers, were recruited and trained on issues related to the community survey, including HIV counselling and testing. All research assistant teams were provided with a questionnaire and Global Positioning System (GPS) devices to capture the coordinates of all the surveyed areas.

All study participants were required to sign an informed consent form before the interview. Research assistant used Swahili to collect the participants’ information. HIV infection was diagnosed using a rapid HIV testing algorithm recommended by the Ministry of Health, as per national comprehensive guidelines on HIV testing services. Testing was conducted with SD Bio-Line®, followed by Trinity Biotech’s (Ireland) Uni-Gold® for confirmation (Testing Algorisms TZ). Research assistants performed pre- and post-counselling before conducting HIV tests on eligible participants. HIV results were voluntarily shared with participants, and those diagnosed with HIV were counselled on antiretroviral initiation and linked to health facilities. For individuals already known to be HIV positive, their HIV status was confirmed with evidence of a Care and Treatment 2 (CTC 2) card.

### Study variables

The primary outcome variable (Dependent Variable) was HIV infection, categorised as a binary variable with values of YES or NO and measured by performing an HIV testing or an interviewer-administered questionnaire. The predictor variables (Independent Variables) included social demographic and risky-sexual behaviour characteristics, such as age, which was treated as a continuous variable. Marital Status was categorised as Single, Widowed/Divorced or Cohabiting/Married. Education Level was categorised as No formal education, Primary education, Secondary education, Higher Education or No Response. The district was categorised as Muleba District, Bukoba UD or Bukoba RD. Condom use in the past 12 months was categorised as Always, sometimes, Never or No response. Consumed alcohol before sex was categorised as a Yes, No or No response. Number of sexual partners was categorised as One, Two, Three- five, More than Five or No response. All predictor variables were measured with an interviewer-administered questionnaire.

### Data management and analysis

A cleaned dataset was analysed using Stata version 17.0 (Stata Corp., College Station, TX). Descriptive statistics were summarised using means (standard deviation) and medians (interquartile range) for continuous variables, and categorical variables were summarised by frequency and proportions and presented by tables and narrations. The prevalence of HIV infection was computed as the number of individuals with HIV infection divided by the total number of study participants who consented to HIV testing and counselling and received HIV results during the study period. A chi-squared test was used to test the association between categorical variables. Logistic regression was employed to identify factors associated with HIV infection. The crude and adjusted odds ratios (OR) with their respective 95% confidence intervals were reported to estimate the magnitude of the association.

Univariate analyses were performed by fitting each explanatory variable against the response variable. Explanatory variables with p-values of <0.05 in the univariate analyses and those considered in the literature as potential confounders and of clinical significance were included in the development of multivariable analyses. Also, multicollinearity between exposures was assessed. Statistical significance was considered for a p-value of less than 0.05.

### Ethical consideration

The study protocol, informed consent for adults, assent for children, questionnaires, and permission to conduct the study was approved by the National Institute of Medical Research (NIMR) in Tanzania under letter No. NIMR/HQ/R.8a/Vol.IX/4557. Written informed consent was obtained from all participants during the survey. Assent was obtained from participants under 18 years of age and countersigned by their guardians or parents. All participants were informed of the study’s benefits and risks. Participants could withdraw from the study at any time without losing the benefits provided, including health education, HIV testing, and referral to a health facility when necessary.

## Results

### Sociodemographic characteristics by districts among fishermen

Overall, 774 fishermen were recruited for this study and enumerated from 10 BMUs with a median age of 31 (IQR: 25–38 years). Of all fishermen, 52.6%, 36.3%, and 11% were from Muleba District, Bukoba Rural District, and Bukoba Urban District, respectively. Nearly a quarter (24.2%) of fishermen were aged 25–29, and only (13.9%) were aged 35–39. More than one-third (38.7%) of fishermen reported living across the shores of Lake Victoria for more than five years. More than half (57.5%) of fishermen had attained primary education. Almost two-thirds of fishermen (65.5%) were cohabiting or married (**Table 1**).

**Table 1:** Sociodemographic characteristics by districts among fishermen on the shore of Lake Victoria, Kagera, Tanzania, 2024 (N = 774).

Characteristics	Bukoba UD n = 86	Bukoba RD n = 281	Muleba District n = 407	Total	p-value
**Age group (years)**					**<0.001**
≤24	14 (16.3%)	30 (10.7%)	102 (25.1%)	146	
25–29	25 (29.1%)	64 (22.8%)	99 (24.3%)	188	
30–34	14 (16.3%)	73 (30.0%)	82 (20.2%)	169	
35–39	8 (9.3%)	47 (16.7%)	53 (13.0%)	108	
40+	25 (29.1%)	67 (23.8%)	71 (17.4%)	163	
**Median age (IQR)**	**31 (25–38)**				
**Marital status**					**<0.001**
Single	17 (19.8%)	65 (23.1%)	134 (32.9%)	216	
Cohabiting/Married	66 (76.7%)	183 (65.1%)	258 (63.4%)	507	
Divorced/Widowed	3 (3.5%)	33 (11.8%)	15 (3.7%)	51	
**Education level**					0.187
No formal education	9 (10.5%)	20 (7.1%)	56 (13.8%)	85	
Primary education	54 (62.8%)	166 (59.1%)	225 (55.3%)	445	
Secondary education	20 (23.2%)	82 (29.2%)	110 (27.0%)	212	
Higher education	3 (3.5%)	13 (4.6%)	16 (3.9%)	32	
**Occupation**					0.166
Fishermen only	50 (58.1%)	156 (55.5%)	255 (62.6%)	461	
Fishermen and Other business	36 (41.9%)	125 (44.5%)	152 (37.4%)	313	
**Time lived at the shore (years)**					**<0.001**
Less than a year	8 (9.3%)	34 (12.1%)	59 (14.5%)	101	
1–2	26 (30.2%)	72 (25.6%)	101 (24.8%)	199	
3–4	20 (23.3%)	40 (14.2%)	114 (28.0%)	174	
5+	32 (37.2%)	135 (48.0%)	133 (32.7%)	300	

### Risky behaviours among fishermen on the shore of Lake Victoria

Overall, 90.2% of fishermen reported inconsistent condom use (never or sometimes). The majority of fishermen (75.6%) reported having more than one sexual partner. Almost half of the fishermen (44.2%) used alcohol before sex, and 98.9% reported having a sexual partner within the last 12 months (**Table 2**).

**Table 2:** Risky behaviours among fishermen on the shore of Lake Victoria, Kagera, Tanzania, 2024 (N = 774).

Characteristics	Bukoba UD	Bukoba RD	Muleba District	Total	p-value
**Had a sexual partner** *					0.656
Yes	84 (97.7%)	278 (98.9%)	402 (98.8%)	764	
No	2 (2.3%)	3 (1.1%)	5 (1.2%)	10	
**Alcohol Use before Sex** *					**0.003**
Yes	35 (40.7%)	147 (52.3%)	160 (39.3%)	342	
No	51 (59.3%)	134 (47.7%)	247 (60.7%)	432	
**Condom use** *					**<0.001**
Always	8 (9.3%)	16 (5.7%)	49 (12.1%)	73	
Sometimes	55 (67.0%)	178 (63.4%)	287 (70.5%)	520	
Never use	20 (23.3%)	79 (28.1%)	51 (12.5%)	150	
No response	3 (3.5%)	8 (2.8%)	20 (4.9%)	31	
**Number of Sexual Partners** *					**0.001**
1	13 (15.1%)	73 (25.9%)	97 (23.8%)	183	
2	31 (36.0%)	71 (25.3%)	160 (39.3%)	262	
3–5	21 (24.4%)	64 (22.8%)	84 (20.6%)	169	
5+	18 (20.9%)	67 (23.8%)	51 (12.5%)	136	
No response	3 (3.6%)	6 (2.2%)	15 (3.8%)	24	

*All variables asked within the last 12 Months.

### Magnitude of uptakes of HIV/AIDs services presented by districts among fishermen

The study estimated the uptake of HIV/AIDS services among fishermen; almost two-thirds (60.9%) of fishermen had ever tested for HIV in the past 12 months before the community survey. Fishermen reported a higher uptake (91.8%) of voluntary medical male circumcision (VMMC), and 77.6% of those living with HIV reported using ART. Moreover, 53.7% reported ever using HIV self-testing before our survey. (**Table 3**).

**Table 3:** Uptake of HIV/AIDS services among fishermen on the shore of Lake Victoria, Kagera, Tanzania, 2024 (N = 774).

Characteristics	Bukoba UD	Bukoba RD	Muleba District	Total	p-value
**HIV testing in the past 12 months.**					0.022
Yes	48 (55.8%)	193 (68.7%)	230 (56.5%)	471 (60.9%)	
No	36 (41.9%)	84 (29.9%)	168 (41.3%)	288 (37.2%)	
No response	2 (2.3%)	4 (1.4%)	9 (2.2%)	15 (1.9%)	
**Ever used an HIV self-test**					0.144
Yes	52 (60.5%)	138 (49.1%)	215 (52.8%)	405 (52.3%)	
No	32 (37.2%)	139 (49.5%)	178 (43.7%)	349 (45.1%)	
Not applicable	2 (2.3%)	4 (1.4%)	14 (3.4%)	20 (2.6%)	
**Circumcised.**					0.077
Yes	84 (97.7%)	253 (90.0%)	374 (91.9%)	711 (91.9%)	
No	2 (2.3%)	28 (10.0%)	33 (8.1%)	63 (8.1%)	
**Current on ART among PLHIV (n = 85).**					0.515
Yes	5 (71.4%)	20 (71.4%)	41 (82.0%)	66 (77.6%)	
No	2 (28.6%)	8 (28.6%)	9 (18.0%)	19 (22.3%)	

### Prevalence of HIV infection among fishermen, by sociodemographic and behavioural risk factors

**Fig 2:**
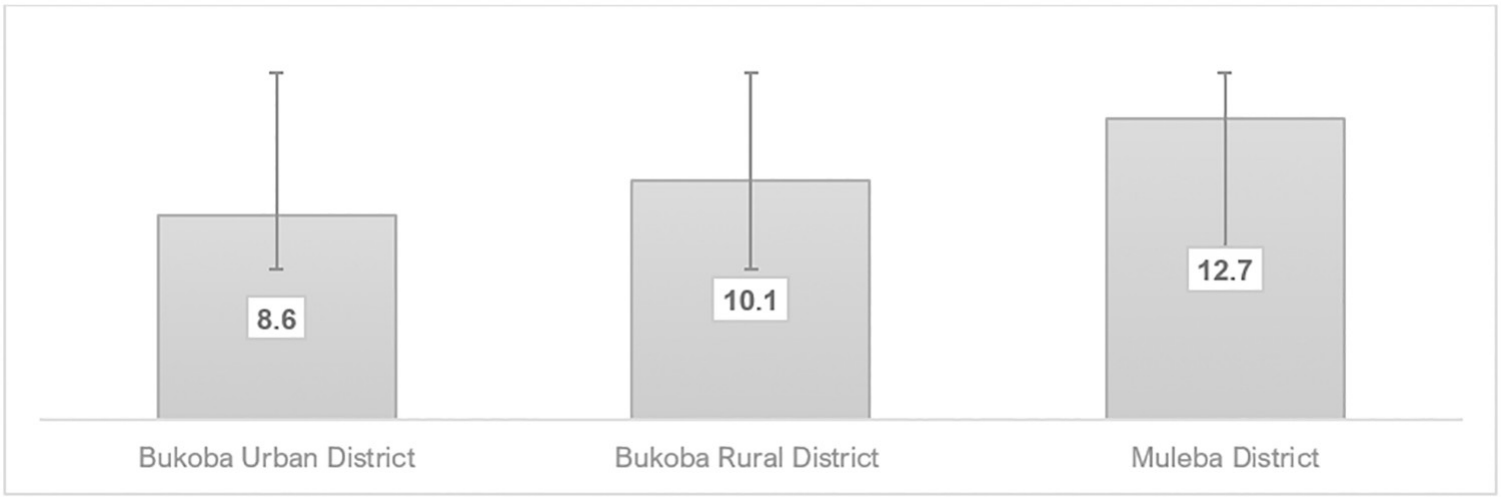
Showing variation of prevalence of HIV infection across the selected districts within Kagera region.

Among 774 fishermen, 752 consented to HIV testing and counselling, having an HIV prevalence of 11.3% (95% CI: 9.2–13.8), with a variation of 8.6% for Bukoba Urban District (UD), 10.1% for Bukoba Rural District (RD), and 12.7% for Muleba District (**Fig 2**). Study participants aged 20–24 years had the highest HIV prevalence (14.7%), and those aged 30–34 years had the lowest prevalence (7.3%). Fishermen with primary education had a higher HIV prevalence (13.4%) compared to others. Most of the fishermen with multiple partners had a higher HIV prevalence compared to those with one sexual partner. Fishermen who did not use condoms during sexual intercourse reported a higher HIV prevalence (16.4%). Additionally, fishermen who used alcohol before sex were estimated to have an HIV prevalence of 14.2% (**Table 4**).

**Table 4:** HIV prevalence by sociodemographic and behavioural risk factors among fishermen in Kagera, Tanzania, 2024 (N = 752).

Characteristics	HIV negative	HIV positive	Total	p-value
**Age group (years)**				0.245
≤24	122 (85.3%)	21 (14.7%)	143	
25–29	160 (89.4%)	19 (10.6%)	179	
30–34	152 (92.7%)	12 (7.3%)	164	
35–39	96 (89.7%)	11 (10.3%)	107	
40+	137 (86.2%)	22 (13.8%)	159	
**Marital status**				0.277
Single	182 (85.6%)	30 (14.2%)	213	
Married/Cohabiting	441 (89.6%)	51 (10.4%)	492	
Divorced/Widowed	44 (91.7%)	4 (8.3%)	48	
**Education level**				**0.006** ^*^
No formal education	80 (97.6%)	2 (2.4%)	82	
Primary education	372 (86.7%)	57 (13.35)	429	
Secondary education	184 (87.6%)	26 (12.4%)	210	
Higher education	31 (100%)	-	31	
**Occupation**				0.932
Fishermen only	397 (88.6%)	51 (11.4%)	448	
Fishermen and other business	270 (88.8%)	34 (11.2%)	304	
**Condom use in the past 12 months**				**0.015** ^*^
Yes	519 (90.3%)	56 (9.7%)	575	
No	148 (83.6%)	29 (16.4%)	177	
**Consumed alcohol before sex**				**0.024** ^*^
Yes	283 (85.8%)	47 (14.2%)	330	
No	384 (91.0%)	38 (9.0%)	422	
**Number of sexual partners**				0.330
1	159 (89.8%)	18 (10.2%)	177	
2	223 (87.4%)	32 (12.6%)	255	
3–5	143 (86.7%)	22 (13.3%)	165	
5+	118 (90.1%)	13 (9.9%)	131	
No response	24 (100%)	-	24	

### Bivariate analysis for factors associated with HIV infections among fishermen

Factors determined to be significant in the crude analysis were those who didn’t use condoms in the past 12 months, consumed alcohol before sex and didn’t test for HIV in the past 12 months. Similar to the crude analysis, the strength and direction of the odds didn’t significantly change in the adjusted model for these variables.

In adjusted analysis, factors that were determined to be significant with higher odds were: no condom use in the past 12 months (1.94 95% CI 1.16–3.27 P = 0.012) compared with those who used condom, did not test for HIV in the past 12 months (4.69 95% CI 2.79–7.88 P<0.001) compared with those who accessed HIV test for the past 12 months, and consuming alcohol before sex (2.32 95% CI 1.42–3.80 P = 0.001) compared with those who don’t consume alcohol before sex. Those with low odds included a lack of formal education (0.09 (95% CI 0.02–0.41, P = 0.002) compared to those with higher education and those aged 30–34 (0.38 (95% CI 0.17–0.85; P = 0.018) compared with those aged 40+ (**Table 5**).

**Table 5:** Bivariate analysis for factors associated with HIV infections among fishermen in Kagera, Tanzania, 2024.

Characteristics	Crude	p-value	Adjusted	p-value
	OR (95% CI)		OR (95% CI)	
**Age group (years)**				
**≤24**	1.07 (0.56–2.04)	0.833	0.81 (0.40–1.65)	0.563
**25–29**	0.74 (0.38–1.42)	0.366	0.79 (0.39–1.63)	0.537
**30–34**	0.49 (0.23–1.03)	0.060	0.38 (0.17–0.85)	0.018
**35–39**	0.71 (0.33–1.54)	0.390	0.62 (0.27–1.39)	0.247
**40+**	1.00		1.00	
**Marital status**				
**Single**	1.81 (0.61–5.41)	0.286		
**Married/Cohabiting**	1.27 (0.44–3.69)	0.657		
**Divorced/Widowed**	1.00		-	
**Education level**				
**No formal education**	0.18 (0.04–0.76)	0.020	0.09 (0.02–0.41)	0.002
**Primary education**	1.08 (0.66–1.78)	0.749	0.69 (0.39–1.21)	0.199
**Secondary education**	-		-	
**Higher education**	1.00		1.00	
**Condom use in the past 12 months**				
**Yes**	1.00		1.00	
**No**	1.82 (1.11–2.95)	0.016	1.94 (1.16–3.27)	0.012
**Consumed alcohol before sex**				
**Yes**	1.68 (1.07–2.64)	0.026	2.32 (1.42–3.80)	0.001
**No**	1.00		1.00	
**District**				
**Bukoba Urban District.**	1.00		-	
**Bukoba Rural District.**	1.19 (0.50–2.83)	0.696		
**Muleba District.**	1.54 (0.67–3.52)	0.310		
**Tested for HIV in the past 12 months**				
**Yes**	1.00		1.00	
**No**	3.65 (2.25–5.89)	<0.001	4.69 (2.79–7.88)	<0.001

## Discussion

This study found a higher HIV prevalence among fishermen along the shores of Lake Victoria at 11.3% compared to the general population. This prevalence is above both the national rate of 4.4% and Kagera’s regional rate of 5.7% [3]. Risky sexual behaviour may be a predictor of the high HIV prevalence, as a meta-analysis study reported that 48% of fishermen tend to have sex with their partners without using condoms [12]. Additionally, the high HIV prevalence may be attributed to the migratory nature of the population due to the seasonality of fish yields and involvement in risky sexual behaviour at various fishing sites [15]. Unswerving with this finding, studies conducted in Uganda [15], and Kenya [19] reported HIV prevalence among fishermen that exceeds 15% compared to the general population. However, hotspot identification and mapping within fishing communities remain significant gaps that are crucial for achieving 95-95-95 targets by 2030. Geographic clustering and hotspot identification can help reveal whether specific locations or subpopulations are receiving insufficient or no HIV services.

Moreover, the study revealed a high prevalence of HIV among fishermen aged 20–24 compared with other age groups, similar to findings from a study conducted in Uganda [15]. This may be due to the fact that many young individuals engage in fishing activities and earn money, which they often use for commercial sex rather than investing in other economic activities. Targeted interventions are needed, such as Social and Behavior Change Communication (SBCC) related to HIV issues, economic strengthening to promote better use of earned money, and increased awareness of HIV prevention services, including condoms, Voluntary Medical Male Circumcision (VMMC), and Pre-exposure Prophylaxis (PrEP).

This study reports risky behaviour related to HIV infection among fishermen, with 85% reporting inconsistent condom use (either never or sometimes), 44.2% consuming alcohol before sex, and more than 90% having multiple sexual partners. Similar findings were reported in studies conducted by Smolak et al [12], and Akobi et al [20], which found risky behaviours such as low condom usage by 48% and alcohol use before sex by more than 40%, respectively. These findings underscore the importance of HIV prevention intervention. Another potential intervention for fishermen to reduce HIV transmission is the use of Pre-exposure Prophylaxis (PrEP). PrEP has been shown to significantly lower the risk of contracting HIV [21] and is available in Tanzania. It can be effective for those at high risk of HIV infection. However, public health challenges for PrEP usage among different subpopulations include demand creation for oral PrEP, the most effective delivery strategies for various demographics and circumstances, the social and behavioural impact of PrEP, and the integration of PrEP services with other services [22]. Modelling based on the HIV pandemic in Kenya suggests that by customising interventions like Voluntary Medical Male Circumcision (VMMC) and PrEP to different patterns of HIV risk, it is possible to prevent up to 600,000 HIV infections by 2030 [23].

Voluntary medical male circumcision (VMMC) can reduce the risk of HIV infection by up to 60% [15]. This study found that VMMC uptake among fishermen was estimated to be more than 90%. Additionally, the study found that fishermen who had sex without a condom had a 94% likelihood of acquiring HIV infection compared to those who used a condom during sexual intercourse. Consistent with this finding, a study by Panga et al [24] found that having sex without a condom had a 1.02 times higher chance of contracting HIV infection. Therefore, consistent condom use should be emphasised among fishermen, even though VMMC services have been widely scaled up in the region. Moreover, a systematic review by WHO found that low condom use is often due to issues related to availability and stigma across communities [25]. Innovations, such as condom vending machines, should be scaled up to increase condom use among high-risk populations, including fishermen. These Condom vending machines are a public health measure to promote safe sex and are often placed in streets, subway stations, and areas with vulnerable populations to HIV infection.

This study also found that alcohol use before sex was significantly associated with HIV infection. Fishermen who reported consuming alcohol before sex had a 2.32 times higher chance of contracting HIV infection compared to those who did not consume alcohol before sex. A similar study by Kapesa et al [10], found that alcohol use before sex had a 2.43 times higher chance of contracting HIV infection. The causality of these findings may include ignorance of the HIV risk associated with excessive alcohol consumption and peer pressure regarding alcohol use before sex. These findings highlight the importance of social and behavioural change communication (SBCC) to promote changes in knowledge, attitudes, norms, beliefs, and behaviour related to HIV prevention among fishermen. Additionally, alcohol consumption before sex contributes to lower condom use, perpetuating HIV infection within fishing communities.

This study revealed that 77.6% of fishermen living with HIV (FLHIV) are on ART. This finding suggests that either fishermen have a low uptake of ART services or that many are unaware of their HIV status. A similar study conducted in Uganda [15] reported that 22.4% of HIV-infected fishermen were not using ART. These findings highlight a gap in case identification or a low uptake of ART services among fishermen and suggest a scale-up of HIV testing interventions such as Index testing and social network testing for early diagnosis and to ensure enrollment in care and treatment.

Moreover, this study found that fishermen who had not accessed HIV testing in the past 12 months were significantly more likely to be HIV infected, with a 4.69 times higher chance of infection. This aligns with findings from Kapesa et al [10] which reported a 2.15 times higher chance of HIV infection among fishermen who had not tested for HIV in the past 12 months. These studies highlight a gap in the fight against HIV among fishermen since the majority of fishermen do not access HIV testing frequently, which increases HIV transmission due to infected individuals not being on ART. Strengthening HIV testing programs for those who have not tested for HIV within the last 12 months is crucial, as recommended by Tanzania HIV Testing Guidelines [26], to reduce HIV late diagnosis. Scale-up HIV self-testing, linkage to care, and treatment for key and vulnerable populations is strongly recommended to meet the 95’ 95’ 95’ goals [27] across the fishing communities.

### Policy implications

Based on the study’s findings, this population should be classified as a key population due to the estimated higher HIV prevalence. Efforts should be made to leverage the influence of fellow fishermen to create demand and increase awareness of HIV services. Both governmental and non-governmental institutions should effectively communicate HIV prevention using Pre-Exposure Prophylaxis (PrEP), as it is among the most effective prevention services. Establishing mobile HIV testing points across fishing communities is crucial, given the lack of frequent testing services. Additionally, community outreach and friendly services will increase awareness and uptake of HIV services.

### Strength and limitations

The study used up-to-date household data among fishermen from various shores of Lake Victoria in the Kagera Region, Tanzania. However, no dataset is complete; the study involved only a few selected BMUs (Beach Management Units) along the shore of Lake Victoria, leaving out several islands with a large number of fishermen who may have high HIV-risk behaviours. Additionally, convenience sampling was used during the data collection period, which may cause potential bias. Therefore, the results should be interpreted with caution.

## Conclusion

Despite a decrease in HIV prevalence to 11.3% from 12% reported in a 2017 study of fishing communities, the estimated prevalence remains high, especially in Muleba District. However, there have been improvements in some HIV prevention services, such as the scale-up of voluntary medical male circumcision (VMMC) and antiretroviral therapy (ART) among fishermen who are aware of their HIV status. Nonetheless, gaps persist, including rates of low HIV testing in the last 12 months and higher HIV prevalence among young people. To address these issues, HIV strategies should focus on young people by enhancing prevention and care efforts, scaling up social and behavioural change communication (SBCC) sessions, and increasing the availability of pre-exposure prophylaxis scale-up across fishing communities.

## Supporting information

S1 Appendix Questionnaire. (DOCX)

## Abbreviations

WHO: World Health Organization
THIS: Tanzania HIV Impact Survey
VMMC: Voluntary Medical Male Circumcision
