# Supplementary material for: Prevalence of HIV infection and uptake of HIV/AIDs services among fishermen on the shores of Lake Victoria in Kagera region, Northwestern Tanzania
Source: PLoS One. 2025 Jan 24;20(1):e0315265. doi: 10.1371/journal.pone.0315265 (PMC11760556; doi:10.1371/journal.pone.0315265)
Supplement: S1 Appendix — (DOCX) [file pone.0315265.s001.docx]

**Appendix 1:** **Questionnaire**

My name is …………………………………... I am conducting a study on the prevalence of HIV Infection, uptake of HIV/AIDS services, and associated risk factors of HIV Infection among fishermen on the shores of Lake Victoria in the Kagera Region.

I kindly request your participation in this study. The findings will help to provide a forward plan for reducing HIV transmission among the fishing communities. The estimated time to complete the questionnaire is not more than 30 minutes.

**Study title: Prevalence of HIV Infection and Uptake of HIV/AIDS Services among Fishermen on the shores of Lake Victoria, in Kagera Region, Tanzania**

District............................................................. Ward...................................................................

Unique code ID.....................................BMU’s...........................................................................

GPS...............................................................................................................................................

| **S/N** | **Area** | **Question** | **Response** |
| --- | --- | --- | --- |
| 1 | **Demographic characteristics** | 1. Age | ................................... |
|  |  | 1. Marital status | 1. Married 2. Single 3. Widowed 4. Divorced 5. Cohabiting 6. Others 7. No response |
|  |  | 1. Education level | 1. No formal education 2. Primary education 3. Secondary education 4. College/University Education 5. No response |
|  |  | 1. Occupation | 1. Fisherman 2. Fisherman and other business 3. No response |
|  |  | 1. Residence | 1. Permanent resident across the shores. 2. Comes for business purposes 3. Others........................... 4. No response |
|  |  | 1. How long have you lived across the shores? | 1. Less than a Year 2. 1-2 Years 3. 3-4 Years 4. 5+ Years 5. No responses |
| **2** | **HIV practice/ uptake of services** | Have you ever happened to have a sexual partner for the past 12 months? | 1. Yes 2. No 3. No response |
|  |  | If Yes, how many sexual partners have you had in the last 12 months? | 1. One 2. Two 3. Three- five 4. More than Five 5. No response 6. Not Applicable (*If answered No*) |
|  |  | Do you use alcohol before sex? | 1. Yes 2. No 3. No responses |
|  |  | When you have been in this working environment for the past 12 months, how often do you use condoms during sex? | 1. Always 2. Sometimes 3. Never 4. No responses |
|  |  | Did you get a chance to be circumcised? | 1. Yes 2. No |
|  |  | For the past 12 months, have you ever tested for HIV? | 1. Yes 2. No 3. No responses |
|  |  | Do you know about HIV Self Testing Services? | 1. Yes 2. No 3. No response. |
|  |  | Have you ever used HIV Self Testing Kits? | 1. Yes 2. No 3. No responses |
| **6** | **HIV Testing** | Would you wish to know your HIV status? | 1. Yes (pretest counselling) 2. No 3. I’m HIV positive (proof?) 4. No response. |
|  |  | HIV screening results | 1. Positive (posttest counselling and referral) 2. Negative 3. Undetermined 4. Unknown 5. Not applicable |
|  |  | If HIV Positive, Are you on Anti retro therapy (ART)? | 1. Yes 2. No 3. No responses |

**How to create a unique code ID:**

Step 1: Identify the first three of the selected districts as described below

- Muleba District -MUD.
- Bukoba Urban District-BUD.
- Bukoba Rural District - BRD

Step 2: Identify the date of Birth, Month and Years of the study participants (05121990)

*Examples of a unique code ID: MUD05121990.*
